# Carry-Over Quality of Pre-acclimatization to Altitude Elicited by Intermittent Hypoxia: A Participant-Blinded, Randomized Controlled Trial on Antedated Acclimatization to Altitude

**DOI:** 10.3389/fphys.2020.00531

**Published:** 2020-05-29

**Authors:** Benedikt Treml, Axel Kleinsasser, Tobias Hell, Hans Knotzer, Maria Wille, Martin Burtscher

**Affiliations:** ^1^Department of General and Surgical Intensive Care Medicine, Medical University Innsbruck, Innsbruck, Austria; ^2^Department of Anesthesiology and Critical Care Medicine, Medical University Innsbruck, Innsbruck, Austria; ^3^Department of Mathematics, Leopold – Franzens University Innsbruck, Innsbruck, Austria; ^4^Department of Anesthesiology and Critical Care Medicine, Klinikum Wels – Grieskirchen, Wels, Austria; ^5^Department of Sport Science, Medical Section, University Innsbruck, Innsbruck, Austria

**Keywords:** acclimatization, normobaric hypoxia, carry-over, intermittent hypoxia, Lake Louise Score

## Abstract

Intermittent normobaric hypoxia (IH) is increasingly used to pre-acclimatize for a sojourn to high altitude. There is a number of hypoxia – protocols observing the hypoxic ventilatory response (HVR), but little is known about the carry – over quality of the Lake Louise Score (LLS). We thus studied a week – long, 1 h per day poikilocapnic hypoxia protocol on whether acclimatization could be carried over for one week. Rationale for this was that it usually takes one week to get from Europe, Britain or the United States to the base camp of a major mountain. Forty-nine healthy volunteers of both sexes were exposed to daily bouts of 1 h at an inspiratory fraction of oxygen (FiO_2_) of 0.11 or 0.21 (control) for 7 consecutive days. Seven days after cessation of IH or sham exposures participants were again subjected to hypoxia (FiO_2_ = 0.11) for 6 h and measurements of isocapnic HVR and blood gases out of the arterialized earlobe were taken and LLS was assessed. In those with IH exposures LLS was reduced which was not the case in those with sham exposure (87 vs. 50%). Changes in HVR or the arterial hemoglobin saturation were not observed. Gender neither affected LLS nor HVR nor blood gases or carry -over quality. We found that our week – long, hypoxia protocol grants a reduction in LLS that can be carried over the time span of one week. In this way, antedated acclimatization may improve safety and comfort on the mountain.

## Introduction

Man has been ascending to altitude for thousands of years and accounts on sickness high up have been there just as long. The Chinese official Too-Kin reported health problems on trading routes from Xinjiang to northern Afghanistan via Pamir, Karakoram and Hindukush mountain passes between the years 37 and 32 BC. Too-Kin reported headache and nausea as the leading complaints back then and headache plus at least another symptom is the definition of acute mountain sickness (AMS) today (Gilbert, 1983; Roach et al., 2018).

AMS is the mildest of all altitude-related illnesses (Luks et al., 2017). AMS usually sets in 1 day after arrival at altitude with symptoms ranging from simple indisposition to actual sickness. However, previous work of Burtscher et al. demonstrated progression of moderately and severely AMS-affected individual only after 6 h (Burtscher et al., 2014).

Diagnosis and classification of AMS severity are made using the Lake Louise score (LLS) worksheet (Hackett and Roach, 2001; Roach et al., 2018). This worksheet evaluates for symptoms such as headache, gastrointestinal problems, fatigue, and lightheadedness and also how these symptoms affect activities at altitude (Roach et al., 2018).

AMS is the most common of all altitude-related conditions. Prevalence in the European Alps depends on the altitude reached: While only 9% of all sojourners are affected at 2,850 m, 34% become sick at 3,650 m and 53% at 4,559 m (Maggiorini et al., 1990). If symptoms do not improve over 2 to 3 days, AMS treatment options include descent and supplemental oxygen. The usual approaches to prevention of AMS include gradual ascent and acetazolamide (Luks et al., 2019).

Recently, the question has come up if it is possible to prepare for altitude at home, so that on the mountain, AMS will not occur at all or at least with fewer symptoms. With this in mind, some tour companies already offer packages which *–* besides guided ascents *–* include intermittent hypoxia (IH) sessions before departing for the journey. The use of IH was shown to increase the hypoxic ventilatory response (Bernardi et al., 2001; Katayama et al., 2001, 2005, 2007; Foster et al., 2005). This will consequently reduce the occurrence of altitude *–* associated illnesses.

Ventilatory acclimatization seems to be the primary mechanism of acclimatization to hypoxia and altitude. Also, short exposures were shown to increase the hypoxic ventilatory response (HVR), lowering the incidence of AMS (Bernardi et al., 2001; Katayama et al., 2001; Foster et al., 2005). Just 1 h per day for 7 days at a simulated altitude of 4,500 m was enough to increase HVR and the arterial oxyhemoglobin saturation (SaO_2_) (Katayama et al., 2001).

In a previous study of ours, a week-long protocol with an hour per day in normobaric hypoxia (equivalent to 4,500 m) did not affect the incidence of AMS but resulted in reduced severity, reflected by a lower LLS score (Wille et al., 2012). Although a noteworthy finding, this study left some questions unanswered: First, this experiment did not include women and gender-specific differences exist. Recently, the menstrual cycle and menopause have been shown to influence the physiological responses to hypoxic exercise (Richalet et al., 2019). Second, the sample size was limited (*n* = 26). Third, the time span between conditioning and testing was only 2 days and it would be interesting to see how long this LLS reduction would last since antedated acclimatization which can be carried over might increase safety on the mountain. Regarding those recent queues near the summit of Mt. Everest, sound acclimatization becomes an even more important factor of preparedness particularly in climbers with lesser mountaineering skills.

Getting to base camps of major mountains in Asia, Africa, Denali or South America from Europe, Britain or the US usually takes a week rather than the 2 days used in our previous study (Wille et al., 2012).

In the light of these manifold influences on acclimatization and development of AMS we sought to clarify the value of pre-acclimatization elicited by a week-long IH protocol. We hypothesized that such an LLS reduction can be carried over in a large sample of either sex.

## Materials and Methods

### Graphic Outline

**FIGURE 1:**
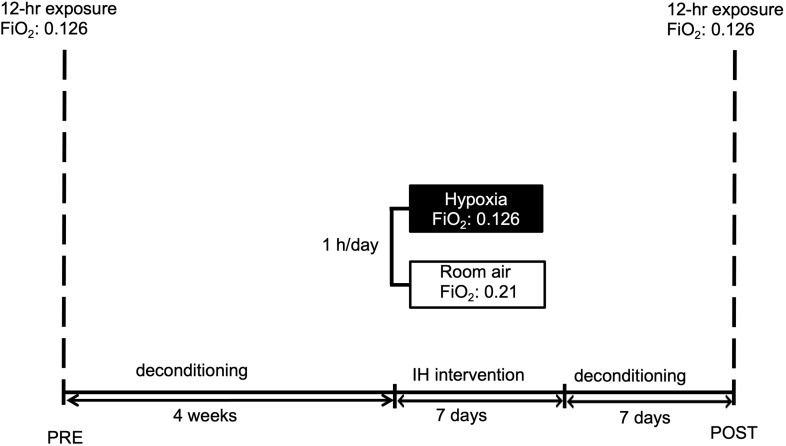
Graphic display of the study schedule. Subjects were familiarized to hypoxia (12 h) followed by a 4-week period of deconditioning. Thereafter short-term pre-acclimatization (1 h for 7 days) or sham-exposure were performed. After 1 week deconditioning the hypoxic exposure (12 h) with measurements followed. IH reflects intermittent hypoxia. FiO_2_ reflects fraction of inspired oxygen.

A graphic outline of the experiment is given in Figure 1.

### Setting

Normobaric hypoxia chamber located in the Department of Sports Science, Leopold-Franzens University, Innsbruck, Austria.

### Study Patients

Seventy-seven healthy female and male subjects were included in the study after a medical check.

Exclusion criteria were permanent residence above 1,000 m, any overnight stay at altitudes above 2,500 m in the previous month or an exposure above 2,500 m two weeks prior to the first tests or during the following investigation period. Any history of lung, cardiac, neurological and psychiatric disease, respectively would have led to exclusion.

Written informed consent was obtained from all participants. The study had been approved by the ethical committee of the Innsbruck Medical University (Protocol Number UN 4522/306/4.11).

### Experimental Design

This study was designed as a participant-blinded, randomized controlled trial. Randomization was performed using an algorithm freely available on the Graph Pad web site^1^ provided by Graph Pad software, 2,365 North Side Drive, Suite 560, 92,108 San Diego.

Participants were subjected to normobaric hypoxia. The oxygen concentration in the hypoxia chamber (Hypoxico OHG, Traunstein, Germany) was set to 11.0% mimicking an altitude of 5,300 m applied in Innsbruck, 575 m. The study was drafted to stay for 8 h (no physical activity) to familiarize with hypoxia and measurement procedures (pre-test).

After a de-acclimatization period of four weeks, subjects were allocated randomly into two groups. One group was exposed to aclimatization using intermittent hypoxia (IH), whereas the control group was treated with room air (FiO_2_ = 0.21). Seven days after cessation of the IH or sham exposures the participants were again subjected to hypoxia (FiO_2_ = 0.11) for 6 h. All participants were encouraged to not consume coffee or alcohol and to refrain from exercising in the 24 h prior to the investigation.

Baseline measurements were performed right before entering the hypoxic chamber. Demographic data, LLS, arterial blood gas analysis, systemic arterial blood pressure and heart rate were also recorded.

Measurements with all parameters were taken at 30 min, 3 and 6 h of continuous hypoxia. Inside the hypoxia chamber air temperature was kept on a constant level of 20°C (72° F). Figure 1 displays the experimental schedule.

### IH – Protocol

Our protocol of short-term acclimatization consisted of daily bouts of 1 h at an FiO_2_ of 0.11 for 7 consecutive days.

Participants in the control group received an FiO_2_ of 0.21 (sham acclimatization, controls) over 1 h for 7 days at FiO_2_ = 0.21. Blinding: When in the chamber, participants could not perceive group assignments (0.11 or 0.21 FiO_2_).

### Data Collection

Hemodynamic parameters were collected with the subjects in sitting position. Pulse-oxymetric oxygen saturation (SpO_2_) measurements were performed with a fingertip pulse-oxymeter (Pulsox-3i Minolta, Osaka, Japan) calculating the mean value over a period of 5 min. Values were obtained before entering hypoxia and after 30 min, 3 and 6 h in hypoxia, respectively. During IH exposure or sham-treatment SpO_2_ was recorded after 25 and 55 min. Heart rate (HR) was monitored with Polar S810, RS800CX (Polar Electro OY, Kempele, Finland).

An arterial blood gas analysis and arterial lactate measurement were performed on every participant at each time point by puncture of the arterialized ear lobe (Miniphotometer plus LP 20; Hach Lange, Berlin, Germany). To exclude hyperventilation we measured ventilation simultaneously.

### Assessment of AMS

The severity of AMS was evaluated by using the LLS: Briefly, participants were asked to rate the symptom complexes headache, gastrointestinal symptoms, fatigue and/or weakness, and dizziness from 0 (not present) to 3 (severe). Summing all items up calculated a total score ranging from 0 to 12 (Roach et al., 2018).

The subjects were classified as having AMS when headache was present and at least one other symptom, with a total score of 4 (Roach et al., 2018).

### Hypoxic Ventilatory Response

HVR was determined using a progressive isocapnic hypoxic test (Wille et al., 2012). After resting for twenty min, the subjects were connected to a re-breathing circuit. Expired minute ventilation (VE) was determined breath by breath, and end tidal partial pressure of CO_2_ (pETCO_2_) (Oxycon Mobile, Jäger, Germany) and SpO_2_ (Pulsox-3i; Minolta, Osaka, Japan) were recorded continuously. PETCO_2_ was held stable by drawing part of the expired air through a CO_2_ absorber. The test was terminated when SpO_2_ dropped to 75%, which took an average of 5 min to do so. HVR was estimated as the slope of the line calculated by linear regression relating changes in VE to SpO_2_ (ΔVE/ΔSpO_2_, L/min%), with slopes presented as positive numbers by convention (Katayama et al., 2009). HVR was assessed before entering the hypoxic chamber at pre-test and post-test.

### Statistical Analysis

Based on the data from Burtscher et al. (2008), who found a reduction of 66.6% in AMS incidence through a short-term acclimatization protocol (1–2 h per day for 5 days at FiO_2_ 15.5–11.0) and an expected AMS incidence of 50% at 4,500 m (Maggiorini et al., 1990), a sample size of 22 patients per group was calculated to assess the primary endpoint with 90% power to detect a difference of 33% in the percentage of patients achieving a LLS reduction of using Fisher’s exact test with 0.05 two-sided significance level.

Main outcome variables of our study were LLS, incidence of AMS, HVR, SpO_2_ and whether or not LLS reduction was still present one week after the hypoxia protocol (i.e., the “carry-over”). Only patients with an assessed LLS at pre- and post-testing were included in the final analyses.

A mathematician not involved in the study procedures or patient assessment (TH) performed the statistical analyses of the results using R, version 3.5.3. All statistical assessments were two-sided and a significance level of 5% was used. The Wilcoxon rank sum test and Fisher’s exact test were applied to assess differences between treatment groups as well as intragroup. We present continuous data as medians (25th*–*75th percentile) and binary variables as number of total. We show effect size and precision with estimated median differences for continuous data and odds ratios (OR) for binary variables, with 95% confidence intervals (CI). In addition, we provide the number needed to treat (NNT) for a carry-over reduction in LLS.

Stratified by groups, the course of the continuous measurements is illustrated by the sequence of the median with corresponding 95% CIs in an exploratory manner for pre- and post-testing.

## Results

Seventy-seven participants were originally included in the study, 49 complete data sets could be obtained. Seventeen participants were excluded for not appearing for the experimental run (*n* = 9), prematurely leaving the chamber (*n* = 5) or incomplete data collection (blood gas analyzer failure, *n* = 4).

Prior to the experiment blood gas variables and biometrics were comparable in both groups (see Table 1).

**TABLE 1:** Characteristics^a^ of experiments.

	Total (*n* = 49)	Acclimatization (*n* = 23)	Control (*n* = 26)	Estimate^b^ with 95% CI	*p*-value^c^
Male gender	25/49 (51.0%)	11/23 (47.8%)	14/26 (53.8%)	1.27(0.36*to*4.53)	0.7775
Age (years)	24.0 (22.0–28.0)	24.0 (22.0–30.0)	24.0 (21.3–27.8)	1(−1*to*4)	0.3394
Height (cm)	174.0 (169.0–180.0)	174.0 (170.0–178.5)	174.0 (168.3–180.0)	0(−5*to*5)	0.8567

*^*a*^Binary data are presented as no./total no. (%), continuous data as medians (25th to 75th percentile). ^*b*^Odds ratios for binary variables and estimated median difference for continuous variables. ^*c*^Assessed by Fisher’s Exact Test for categorical variables and Wilcoxon Rank Sum Test for continuous variables in intergroup comparison. CI reflects confidence interval.*

Arterial pH increased during acute hypoxia with no difference between the sham and real IH exposure groups (see Table 2). Arterial partial pressures of oxygen (paO_2_) and oxyhemoglobin saturation (SpO_2_) decreased during acute hypoxia but were not different between sham and real IH exposure groups. Arterial partial pressures of carbon dioxide (paCO_2_) decreased during acute hypoxia but was not significantly different between sham and real IH exposure groups except for a trend toward a lower paCO_2_ in acclimatized subjects after 30 min in post-testing. HVR remained unchanged during the course of the experiment and was not different between groups (Table 2).

**TABLE 2:** Blood gas analyses at base line and during hypoxia exposure^a^.

	Total (*n* = 49)	Acclimatization (*n* = 23)	Control (*n* = 26)	Estimate^b^ with 95% Cl	*p*-value^c^
**Ph**					
Pre-testing: baseline	7.41(7.39−7.43)	7.41(7.39−7.43)	7.41(7.39−7.42)	0.00(−0.02to0.02)	1
Pre-testing: 30 min	7.43(7.41−7.45)	7.43(7.41−7.47)	7.43(7.42−7.44)	0.01(−0.01to0.04)	0.5175
Pre-testing: 360 min	7.46(7.44−7.48)	7.45(7.44−7.48)	7.46(7.45−7.48)	0.00(−0.03to0.02)	0.8756
Post-testing: baseline	7.39(7.38−7.42)	7.41(7.38−7.44)	7.39(7.37−7.41)	0.02(−0.01to0.05)	0.2108
Post-testing: 30 min	7.43(7.41−7.46)	7.45(7.41−7.5)	7.43(7.41−7.44)	0.02(−0.01to0.07)	0.2124
Post-testing: 360 min	7.46(7.44−7.47)	7.46(7.44−7.49)	7.46(7.44−7.47)	0.01(−0.02to0.04)	0.5618
**Pa0_2_(mmHg)**					
Pre-testing: baseline	78(73−82)	76.0(73.8−79.3)	80(73−84)	−2(−8to3)	0.4377
Pre-testing: 30 min	38(34−1)	35.5(33.8−38.3)	39(34−2)	−2(−7to2)	0.2657
Pre-testing: 360 min	38(35−2)	36.0(35.0−39.0)	41(37−3)	–3 (–7 to 1)	0.1686
Post-testing: baseline	77(73−82)	75.5(72.5−78.8)	78(74−82)	−2(−6to3)	0.3626
Post-testing: 30 min	38(37−43)	41.0(37.3−44.3)	38(37−41)	2 (–2 to 7)	0.2052
Post-testing: 360 min	38(36−2)	39.0(36.8−4.0)	38(35−40)	2(−2to6)	0.3860
**Sp0_2_ (%)**					
Pre-testing: baseline	98.0(97.0−98.0)	98.0(96.5−98.0)	98.0(97.0−99.0)	–1 (–2 to 1)	0.2938
Pre-testing: 30 min	80.8(78.4−84.3)	80.0(79.0−88.5)	81.0(78.0−84.0)	1.4 (–2.6 to 7.2)	0.5426
Pre-testing: 360 min	81.4 (79.2 – 87.7)	81.2(79.3−85.1)	81.5(76.8−87.7)	0.6(−5.9to6.1)	0.9077
Post-testing: baseline	97.5(97.0−98.0)	97.0(96.5−97.5)	98.0(97.0−98.0)	−1(−1to0)	0.0633
Post-testing: 30 min	83.2(78.5−87.0)	86.1(81.5−90.6)	80.7(78.0−84.8)	4.1(−0.7to9.6)	0.1240
Post-testing: 360 min	83.8(78.7−85.4)	84.5(79.7−89.3)	83.3(78.6−84.8)	1.4(−3.8to7.6)	0.5623
PaC0_2_(mmHg)					
Pre-testing: baseline	37.5(34.8−0.0)	37.0(35.0−0.5)	38(34−39)	1(−3to4)	0.8161
Pre-testing: 30 min	35.5(33.0−38.0)	34.0(32.5−38.0)	36(34−38)	–1 (–4 to 2)	0.5211
Pre-testing: 360 min	32.5(30.8−36.0)	32.0(32.0−35.5)	33(30−36)	0(−3to3)	0.9303
Post-testing: baseline	38.0(36.0−1.3)	37.0(34.5−41.5)	39(36−41)	−2(−5to2)	0.5599
Post-testing: 30 min	34.5(32.0−38.0)	32.0(29.0−35.5)	37(33−38)	−4(−8to0)	0.0515
Post-testing: 360 min	33.5(29.8−35.5)	33.0(28.5−34.0)	34(32−37)	−2(−6to2)	0.2557
**HVR (L/min%)**					
Pre-testing	0.64(0.36−1.01)	0.74(0.50−1.03)	0.44(0.33−0.96)	0.22(−0.03to0.48)	0.0987
Post-testing	0.60(0.31−0.89)	0.61(0.30−0.87)	0.59(0.35−0.87)	−0.03(−0.29to0.23)	0.8194

*^*a*^Binary data are presented as no./total no. (%), continuous data as medians (25th to 75th percentile). ^*b*^Odds ratios for binary variables and estimated median difference for continuous variables. ^*c*^Assessed by Fisher’s Exact Test for categorical variables and Wilcoxon Rank Sum Test for continuous variables in intergroup comparison. Cl reflects confidence interval, pH reflects arterial pH, pa0_2_ reflects arterial partial pressure of oxygen taken from the arterialized earlobe, Sp0_2_ reflects peripheral saturation of oxygen, paC0_2_ reflects arterial partial pressure of carbon dioxide taken from the arterialized earlobe, HVR reflects hypoxic ventilatory response.*

**FIGURE 2:**
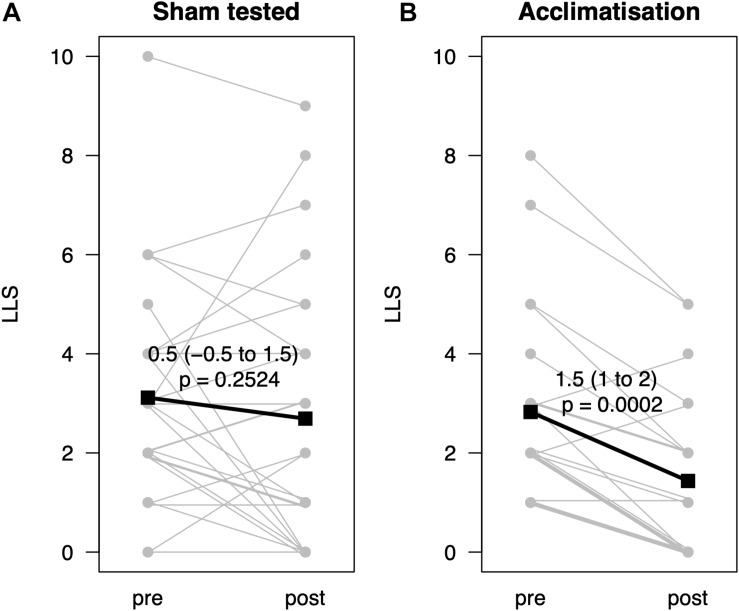
Change of Lake Louise Scores before and after hypoxic exposure (*n* = 49) with or without acclimatization. **(A)** Displays the before-and-after Lake Louise Scores (LLS, y-axis) of those with a sham treatment, **(B)** of those with a real intermittent hypoxia. Data represented as estimated median difference with 95% CIs (bold). *P*-values assessed by Wilcoxon signed rank test. For clarity single lines may represent several subjects.

**FIGURE 3:**
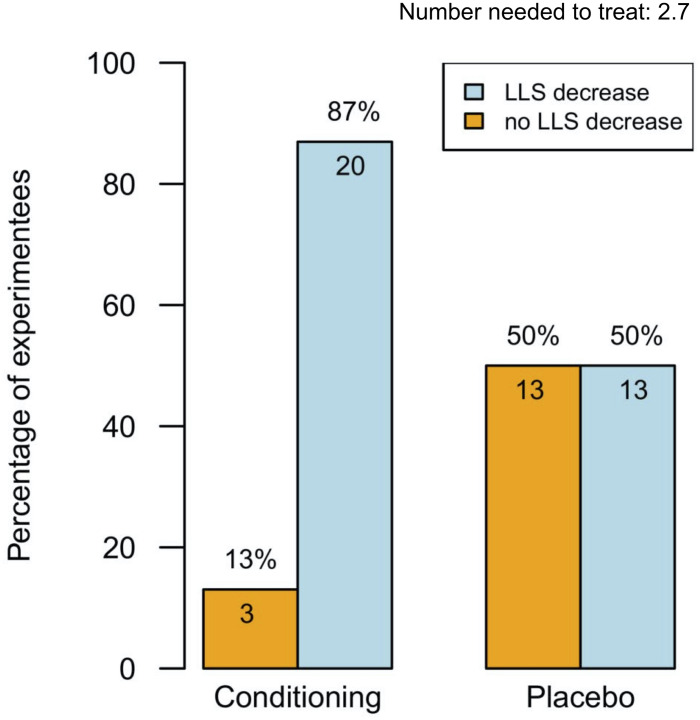
Percentages of experiments (*n* = 49) with and without Lake Louise Score decrease. This figure displays the effectiveness of intermittent hypoxia conditioning (blue boxes) or sham treatment (yellow boxes) on the Lake Louise Score. Data represented as percentage. Number needed to treat for a carry-over reduction in Lake Louise Score: 2.7.

**FIGURE 4:**
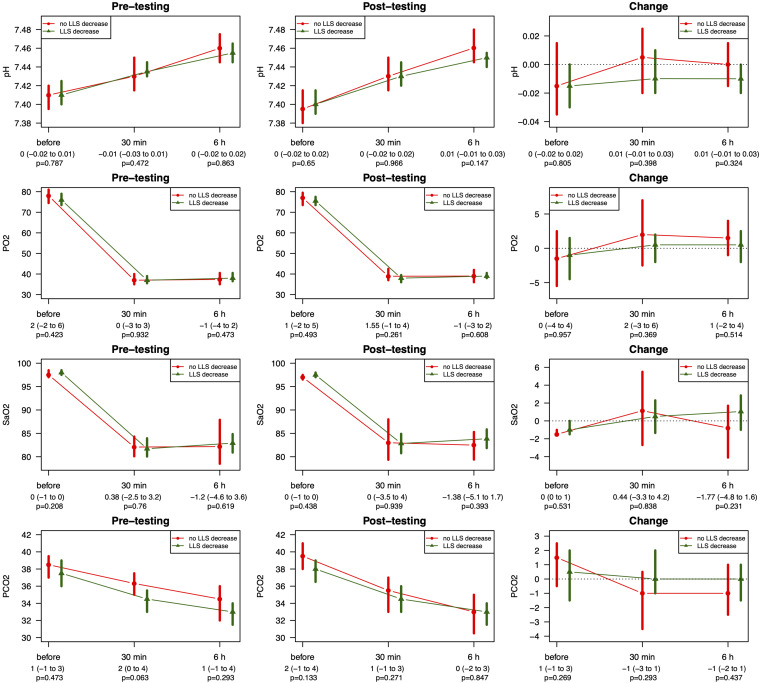
Course of pH, SpO_2_, and paCO_2_ stratified by LLS decrease for pre- and post-testing pH reflects arterial pH, paO_2_ reflects arterial partial pressure of oxygen taken from the arterialized earlobe, SpO_2_ reflects peripheral saturation of oxygen, paCO_2_ reflects partial pressure of carbon dioxide taken from the arterialized earlobe. Data stratified by LLS decrease are represented as median with corresponding 95% CIs. *P*-values assessed by Wilcoxon rank sum test.

From pre- to post-testing, there was a greater decrease in LLS in the pre-conditioned volunteers than in sham-tested subjects (Figure 2). Figures 2A,B display the median differences of the before- and after-Lake Louise Scores of those who received the sham treatment and those who received the real intermittent hypoxia intervention, respectively. Moreover, 87% percent of the pre-conditioned volunteers had lower LLS while only 50% of sham *–* treated participants showed a reduction in symptom severity (see Figure 3). Moreover, the number needed to treat with respect to carry *–* over LLS reduction was 2.7. The course of pH, paO_2_, SpO_2_, and paCO_2_ stratified by LLS decrease (for both groups combined) are depicted in Figure 4. There were no differences in the pre- to post-testing changes in these variables between those that did and did not show a decrease in LLS upon post-testing.

In summary, those with real IH preconditioning showed reduced LLS despite an unchanged HVR. Moreover, we did not detect an association between LLS decrease and increase of ventilation. No difference between sexes could be observed (data not shown).

## Discussion

In this experiment we evaluated the carry *–* over quality of a hypoxia pre-acclimatization protocol known to lower LLS. After a week-long pre-acclimatization with one daily hour in hypoxia 49 volunteers had a hypoxia-free interval of one week and were then again subjected to hypoxia (FiO_2_ = 0.11). LLS performed then revealed that 87% percent of the pre-conditioned volunteers had lower LLS while only 50% of sham *–* treated participants showed a reduction in symptom severity. The number needed to treat with respect to carry *–* over LLS reduction was 2.7. The reduction in LLS was neither associated with an increase in HVR nor with a rise in SpO_2_. This minor relationship between change of HVR and change of LLS is in line with previous work of our group (Burtscher et al., 2012).

Regarding LLS, acclimatization to hypoxia is possible using week *–* long, 1 h per day protocol and can be carried over for one week. However, the concept of antedated acclimatization is not new: Already in 1992, Richalet and coworkers presented an interesting experiment on pre-acclimatization before climbing Mount Everest (Richalet et al., 1992). Using a hypobaric chamber and actual altitude exposure the athletes examined were found to have no change in ventilator response (Richalet et al., 1992). Still, SpO_2_ was increased on the mountain (Richalet et al., 1992). Obviously, from the viewpoint of the climber, feeling comfortable defines acclimatization better than changes in HVR or SpO_2_.

Traditionally, acclimatization is understood as an individual’s adaption to a change in the environment. In the case of high altitude, acclimatization particularly means tolerance to hypoxia. Taking into account that the volunteers with previous real IH had a lower LLS (i.e., they felt better) than those with sham IH is compatible with the notion of acclimatization although physiological variables were not different between groups.

Potential mechanisms for the development of AMS are outlined by Wilson and coworkers in a recent review on brain, high altitude and AMS: Hypoxemia leads to vasodilatation and hypocarbia leads to vasoconstriction and both are in a delicate balance governed by the HVR (Wilson et al., 2009). Also, nitric oxide, adenosine, and potassium play a role in the tight fit hypothesis, where the hypoxic brain has an increased volume and is then more tightly fitted into meninges causing headache (Wilson et al., 2009). The tight fit hypothesis is yet to be corroborated or rejected, however, it seems credible particularly since nitric oxide is involved: Nitroglycerine as a drug acts via the cGMP pathway and may cause severe headache. Prolonged administration of nitroglycerine frequently shows a reduction in the effectiveness – i.e., reduced vasodilatation – resulting in less vasodilating and thus volume increasing action on the brain. Such a tolerance may play a role in the acclimatization we observe. Only mild increases in brain volume have been associated with acute mountain sickness (Kallenberg et al., 2007). Furthermore, other underlying mechanisms, such as nitric oxide, still need to be elucidated. Moreover, expiratory nitric oxide and brain volume need to be measured to support this notion.

The full scope of symptoms of acute mountain sickness may develop only after 24 h. However, previous work of Burtscher et al. demonstrated progression of moderately and severely AMS-affected individual (most of all headache) only after 6 h (Burtscher et al., 2014). Moreover, we chose this period due to practicability as we were not able letting our subjects sojourn for a day or so.

Taking gender into account may further elucidate the underlying mechanisms of AMS as there is a relationship between incidence of AMS and gender: It was found that women do have a higher HVR due to the respiratory stimulant nature of progesterone (Regensteiner et al., 1989). Moreover, HVR at exercise has been shown to be higher in the second half of the menstrual cycle (Richalet et al., 2019). A higher HVR may be associated with a smaller incidence of AMS in women. In an interesting study an association between sex and incidence of AMS was found only in those women not taking acetazolamide as prophylaxis (Basnyat et al., 2000). However, in this study, not the LLS, but the Hackett’s score was used (Basnyat et al., 2000). Nevertheless, other researchers found a strong association between gender and AMS incidence, like Santantonio and coworkers (Santantonio et al., 2014). Recently, Richalet et al. found similar prevalences of severe AMS (defined as headache and LLS ≥ 6) in pre- and post-menopausal women. In our study, we could not detect any association between sex and the carry *–* over quality of acclimatization.

We calculated the number needed to treat for IH preconditioning/LLS carry-over. The number needed to treat in this context is the number of IH treated volunteers needed to find a carry-over reduction in LLS. The number needed to treat was 2.7 in those exposed to real IH rendering our weeklong, 1 h per day protocol as effective: Eighty-seven percent showed a reduction in LLS (Figure 3). In comparison, acetazolamide (Diamox) 125 mg BID for the prophylaxis of AMS resulted in a number needed to treat of eight, meaning acetazolamide in a comparable setting seems considerably less effective (Basnyat et al., 2003).

A revised Lake Louise score has been presented in 2018 and the consensus group has eliminated sleep disturbances as an AMS symptom (Roach et al., 2018). Our experiment has been performed using the older LLS, however, sleep was not an issue of our 6 h experimental runs.

Using normobaric hypoxia is a possible limitation. Hypobaric hypoxia may yield different results, as outlined in the review of Fulco and coworkers (Fulco et al., 2013). However, hypobaric chambers are less common and consequently there is less research performed in these.

Obtaining blood samples by puncture of the arterialized ear lobe as a surrogate for arterial blood is a limitation and was chosen for being less invasive for our healthy participants.

In an experiment we performed 4 years before, this protocol did not prevent AMS in AMS *–* susceptible climbers (Faulhaber et al., 2016). We repeated this experiment in a larger cohort without this limitation since in AMS *–* susceptible climbers there may be an individual anticipation of protocol efficiency. And this may affect LLS assessments. It was thus also a goal of ours to examine a population without known limitations in hypoxia.

The efficacy of most intermittent hypoxia protocols seems to depend on the enhancement of the hypoxic ventilatory drive which will result in a higher SpO_2_. In this experiment, however, HVR and SpO_2_ did not change, still LLS dropped. As mentioned above, nitric oxide and the cGMP pathway may play a role. As we expected a raised HVR (which we theorized had carry *–* over qualities), we did not plan on taking measurements of exhaled nitric oxide.

Future research may focus on how acetazolamide plus IH preconditioning will affect AMS incidence and LLS. Furthermore, it would be interesting to see if antedated acclimatization will also affect cognitive function, sleep quality or athletic performance.

Another application may be the pre-acclimatization of high altitude pulmonary edema *–* HAPE *–* susceptible climbers. The efficacy of antedated acclimatization in this population also requires investigation.

In our participant-blinded, randomized, controlled trial we demonstrated that antedated acclimatization enables to carry *–* over a LLS reduction. Using a week-long pre-acclimatization with one daily hour in hypoxia revealed a number needed to treat of 2.7. In summary, an acclimatization protocol using intermittent hypoxia seems to be a promising tool in attenuating AMS. Clearly, the significance of such a protocol still has to be further evaluated, especially in groups like AMS- or HAPE *–* susceptible climbers.

## Data Availability Statement

The datasets generated for this study are available on request to the corresponding author.

## Ethics Statement

The studies involving human participants were reviewed and approved by Ethical Committee, Medical University Innsbruck. The patients/participants provided their written informed consent to participate in this study.

## Author Contributions

BT was the investigator in the laboratory and wrote essential sections of the manuscript. AK participated in all laboratory tests, performed literature research and manuscript writing and assembly. TH performed all calculations. HK wrote sections of the manuscript. MW participated in the lab testing. MB was the principal investigator created the experiment’s design and supervised all testing. All authors contributed to manuscript revision, read and approved the submitted version.

## Conflict of Interest

The authors declare that the research was conducted in the absence of any commercial or financial relationships that could be construed as a potential conflict of interest.

## Footnotes

1 https://www.graphpad.com/quickcalcs/randMenu/

## References

[B1] Basnyat B. Gertsch J. Johnson E. Castro-Marin F. Inoue Y. Yeh C. (2003). Efficacy of low-dose acetazolamide (125 mg BID) for the prophylaxis of acute mountain sickness: a prospective, double-blind, randomized, placebo-controlled trial. *High Alt. Med. Biol.* 4 45–52. 12713711 10.1089/152702903321488979

[B2] Basnyat B. Subedi D. Sleggs J. Lemaster J. Bhasyal G. Aryal B. (2000). Disoriented and ataxic pilgrims: an epidemiological study of acute mountain sickness and high-altitude cerebral edema at a sacred lake at 4300 m in the Nepal Himalayas. *Wilderness Environ. Med.* 11 89–93. 10921358 10.1580/1080-6032(2000)011[0089:daapae]2.3.co;2

[B3] Bernardi L. Passino C. Serebrovskaya Z. Serebrovskaya T. Appenzeller O. (2001). Respiratory and cardiovascular adaptations to progressive hypoxia; effect of interval hypoxic training. *Eur. Heart J.* 22 879–886. 11350098 10.1053/euhj.2000.2466

[B4] Burtscher M. Brandstätter E. Gatterer H. (2008). Preacclimatization in simulated altitudes. *Sleep Breath.* 12 109–114. 10.1007/s11325-007-0127-9 18030513

[B5] Burtscher M. Mairer K. Wille M. Gatterer H. Ruedl G. Faulhaber M. (2012). Short-term exposure to hypoxia for work and leisure activities in health and disease: which level of hypoxia is safe? *Sleep Breath.* 16 435–442. 21499843 10.1007/s11325-011-0521-1

[B6] Burtscher M. Wille M. Menz V. Faulhaber M. Gatterer H. (2014). Symptom progression in acute mountain sickness during a 12-hour exposure to normobaric hypoxia equivalent to 4500 m. *High Alt. Med. Biol.* 15 446–451. 10.1089/ham.2014.1039 25341048

[B7] Faulhaber M. Pocecco E. Gatterer H. Niedermeier M. Huth M. Dünnwald T. (2016). Seven passive 1-h hypoxia exposures do not prevent AMS in susceptible individuals. *Med. Sci. Sports Exerc.* 12 2563–2570. 10.1249/mss.0000000000001036 27414687

[B8] Foster G. McKenzie D. Milsom W. Sheel A. (2005). Effects of two protocols of intermittent hypoxia on human ventilatory, cardiovascular and cerebral responses to hypoxia. *J. Physiol.* 567 689–699. 10.1113/jphysiol.2005.091462 15975977 PMC1474187

[B9] Fulco C. Beidleman B. Muza S. (2013). Effectiveness of preacclimatization strategies for high-altitude exposure. *Exerc. Sport Sci. Rev.* 41 55–63. 10.1097/JES.0b013e31825eaa33 22653279

[B10] Gilbert D. (1983). The first documented report of mountain sickness: the China or Headache Mountain story. *Respir. Physiol.* 52 315–326. 10.1016/0034-5687(83)90088-9 6351209

[B11] Hackett P. Roach R. (2001). High-altitude illness. *N. Engl. J. Med.* 345 107–114. 11450659 10.1056/NEJM200107123450206

[B12] Kallenberg K. Bailey D. Christ S. Mohr A. Roukens R. Menold E. (2007). Magnetic resonance imaging evidence of cytotoxic cerebral edema in acute mountain sickness. *J. Cereb. Blood Flow Metab.* 27 1064–1071. 17024110 10.1038/sj.jcbfm.9600404

[B13] Katayama K. Fujita H. Sato K. Ishida K. Iwasaki K. Miyamura M. (2005). Effect of a repeated series of intermittent hypoxic exposures on ventilatory response in humans. *High Alt. Med. Biol.* 6 50–59. 10.1089/ham.2005.6.50 15772500

[B14] Katayama K. Ishida K. Iwasaki K. Miyamura M. (2009). Effect of two durations of short-term intermittent hypoxia on ventilatory chemosensitivity in humans. *Eur. J. Appl. Physio.* 105 815–821. 10.1007/s00421-008-0960-y 19125287

[B15] Katayama K. Sato K. Hotta N. Ishida K. Iwasaki K. Miyamura M. (2007). Intermittent hypoxia does not increase ventilation at simulated moderate altitude. *Int. J. Sports Med.* 28 480–487. 10.1055/s-2006-955895 17357965

[B16] Katayama K. Sato Y. Morotome Y. Shima N. Ishida K. Mori S. (2001). Intermittent hypoxia increases ventilation and SaO2 during hypoxic exercise and hypoxic chemosensitivity. *J. Appl. Physiol.* 90 1431–1440. 11247944 10.1152/jappl.2001.90.4.1431

[B17] Luks A. Auerbach P. Freer L. Grissom C. Keyes L. McIntosh S. (2019). Wilderness medical society practice guidelines for the prevention and treatment of acute altitude illness: 2019 update. *Wilderness Environ. Med.* 25(4 Suppl) S4–S14. 10.1016/j.wem.2019.04.006 25498261

[B18] Luks A. Swenson E. Bärtsch P. (2017). Acute high-altitude sickness. *Eur. Respir. Rev.* 26:160096. 10.1183/16000617.0096-2016 PMC9488514 28143879

[B19] Maggiorini M. Bühler B. Walter M. Oelz O. (1990). Prevalence of acute mountain sickness in the Swiss Alps. *BMJ* 301 853–855. 10.1136/bmj.301.6756.853 2282425 PMC1663993

[B20] Regensteiner J. Woodard W. Hagerman D. Weil J. Pickett C. Bender P. (1989). Combined effects of female hormones and metabolic rate on ventilatory drives in women. *J. Appl. Physiol.* 66 808–813. 10.1152/jappl.1989.66.2.808 2540141

[B21] Richalet J. Bittel J. Herry J. Savourey G. Le Trong J. Auvert J. (1992). Use of a hypobaric chamber for pre-acclimatization before climbing Mount Everest. *Int. J. Sports Med.* 13(Suppl. 1) S216–S220. 1483780 10.1055/s-2007-1024644

[B22] Richalet J. Lhuissier F. Jean D. (2019). Ventilatory response to hypoxia and tolerance to high altitude in women: influence of menstrual cycle, oral contraception, and menopause. *High Alt. Med. Biol.* 21 12–19. 31855465 10.1089/ham.2019.0063

[B23] Roach R. Hackett P. Oelz O. Baertsch P. Luks A. MacInnis M. (2018). The 2018 lake louise acute mountain sickness score. *High Alt. Med. Biol.* 19 4–6. 29583031 10.1089/ham.2017.0164 PMC6191821

[B24] Santantonio M. Chapplain J. Tattevin P. Leroy H. Mener E. Gangneux J. (2014). Prevalence of and risk factors for acute mountain sickness among a cohort of high-altitude travellers who received pre-travel counselling. *Travel Med. Infect. Dis.* 12 534–540. 10.1016/j.tmaid.2014.08.004 25224954

[B25] Wille M. Gatterer H. Mairer K. Philippe M. Schwarzenbacher H. Faulhaber M. (2012). Short-term intermittent hypoxia reduces the severity of acute mountain sickness. *Scand. J. Med. Sci. Sports* 22 e79–e85. 10.1111/j.1600-0838.2012.01499.x 22853822

[B26] Wilson M. Newman S. Imray C. (2009). The cerebral effects of ascent to high altitudes. *Lancet Neurol.* 8 175–191. 10.1016/S1474-4422(09)70014-6 19161909

